# An evaluation of the reliability of muscle fiber cross-sectional area and fiber number measurements in rat skeletal muscle

**DOI:** 10.1186/1480-9222-15-6

**Published:** 2013-03-08

**Authors:** Lisa Ceglia, Sathit Niramitmahapanya, Lori L Price, Susan S Harris, Roger A Fielding, Bess Dawson-Hughes

**Affiliations:** 1 Division of Endocrinology, Diabetes, and Metabolism, Tufts Medical Center, Washington Street, Box 268, 800, Boston, MA, 02111, USA; 2 Jean Mayer U.S. Department of Agriculture, Human Nutrition Research Center on Aging at Tufts University, Boston, MA, 02111, USA; 3 Department of Medicine, Rajavithi Hospital, College of Medicine, Rangsit University, Bangkok, Thailand; 4 Biostatistics Research Center, Institute for Clinical Research and Health Policy Studies, Tufts Medical Center, Box 063, 800, Washington Street, Boston, MA, 02111, USA

**Keywords:** Skeletal muscle, Muscle fiber cross-sectional area, Fiber number, Myofibrillar ATPase activity

## Abstract

**Background:**

The reliability of estimating muscle fiber cross-sectional area (measure of muscle fiber size) and fiber number from only a subset of fibers in rat hindlimb muscle cross-sections has not been systematically evaluated. This study examined the variability in mean estimates of fiber cross-sectional area as a function of the number of fibers measured, and tested whether counting a subset of fibers in a cross-section could predict total fiber number in middle-aged rats.

**Results:**

Soleus and extensor digitorum longus (EDL) muscle cross-sections from 23-month-old, male Fisher 344 x Brown Norway rats were stained for myofibrillar ATPase activity to identify muscle fiber type (either type I [slow-twitch] or II [fast-twitch]) and laminin to facilitate fiber cross-sectional measurements. We outlined the circumference of 1000 to 1600 single muscle fibers for measurement of fiber cross-sectional area within muscle sections. Mean type I fiber cross-sectional area was based on soleus muscle sections which were predominantly composed of type I muscle fibers. Mean type II fiber cross-sectional area was based on EDL muscle sections which were predominantly composed of type II muscle fibers. A bootstrapping resampling technique demonstrated that variability in sampling distribution of mean type I and II fiber cross-sectional areas decreased and gradually stabilized as the number of fibers measured increased with large declines in variability occurring at numbers below 150 fibers. Coefficients of variation for bootstrapped mean type I fiber cross-sectional areas were lower than for type II. In the same muscle sections, total fiber number was compared to fiber numbers within 1, 2, 3, and 4 fixed field areas (10x magnification; 1000 x 1500 pixels in size/field) on the cross-section. Fiber numbers from 3 to 4 fields (approximating 15 to 20% of the cross-section) provided a reasonably predictive value of total fiber number (*r*=0.57-0.59, P=0.003).

**Conclusions:**

These data describe a pattern of improved precision in estimating mean fiber cross-sectional area as sample size of fibers measured increases to at least 150 in this rat model. Counting 15-20% of the fibers in cross-sections provides a reasonably reliable estimate of the total fiber number.

## Background

The study of skeletal muscle atrophy and hypertrophy in the laboratory rat commonly involves 1) the assessment of single muscle fiber size, referred to as the cross-sectional area of skeletal muscle fibers (FCSA), and 2) the number of single muscle fibers in a muscle cross-section (FN). These measurements are performed by experienced operators visually analyzing successive histological muscle cross-sections which contain hundreds to thousands of single muscle fibers. This work also involves the careful identification and evasion of areas distorted by freezing, sectioning, processing, or staining which may affect the accuracy of FCSA and FN measurements. Thus, as these procedures are time-consuming and labor-intensive, they can result in higher costs to conduct the research. What many research groups have done is to estimate FCSA and/or FN based on a representative subset of single muscle fibers within a muscle cross-section [1-11]. Notably, this representative subset can vary from as few as 25 to as many as hundreds of fibers. However, the reliability of estimating FCSA and FN from only subsets of fibers in rat hindlimb muscle cross-sections has not been systematically evaluated.

The objective of this study was to inform decisions about how many fibers to measure in order to obtain a reliable estimate of FCSA and FN. In this report, we first identified the variability in mean estimates of FCSA as a function of the number of fibers measured on hindlimb muscle cross-sections, and secondly evaluated whether counting a subset of fibers within cross-sections could predict total cross-sectional FN. These studies were conducted in twenty-three, male Fisher 344 x Brown Norway F1 hybrid (F344BN) rats. Soleus and extensor digitorum longus (EDL) muscle cross-sections were stained for myofibrillar ATPase activity to identify fiber type (either type I [slow-twitch] or II [fast-twitch]) and laminin to facilitate FCSA measurements. A bootstrapping resampling technique was employed to demonstrate the variability in sampling distribution of mean type I and II FCSAs. Mean type I muscle FCSA was based on soleus muscle sections which were predominantly composed of type I muscle fibers. Mean type II muscle FCSA was based on EDL muscle sections which were predominantly composed of type II muscle fibers. In the same muscle cross-sections, total FN was compared to FN within 1, 2, 3, and 4 fixed field areas (10x magnification; 1000 x 1500 pixels in size/field) on the cross-section.

## Results

### FCSA

**Figure 1:**
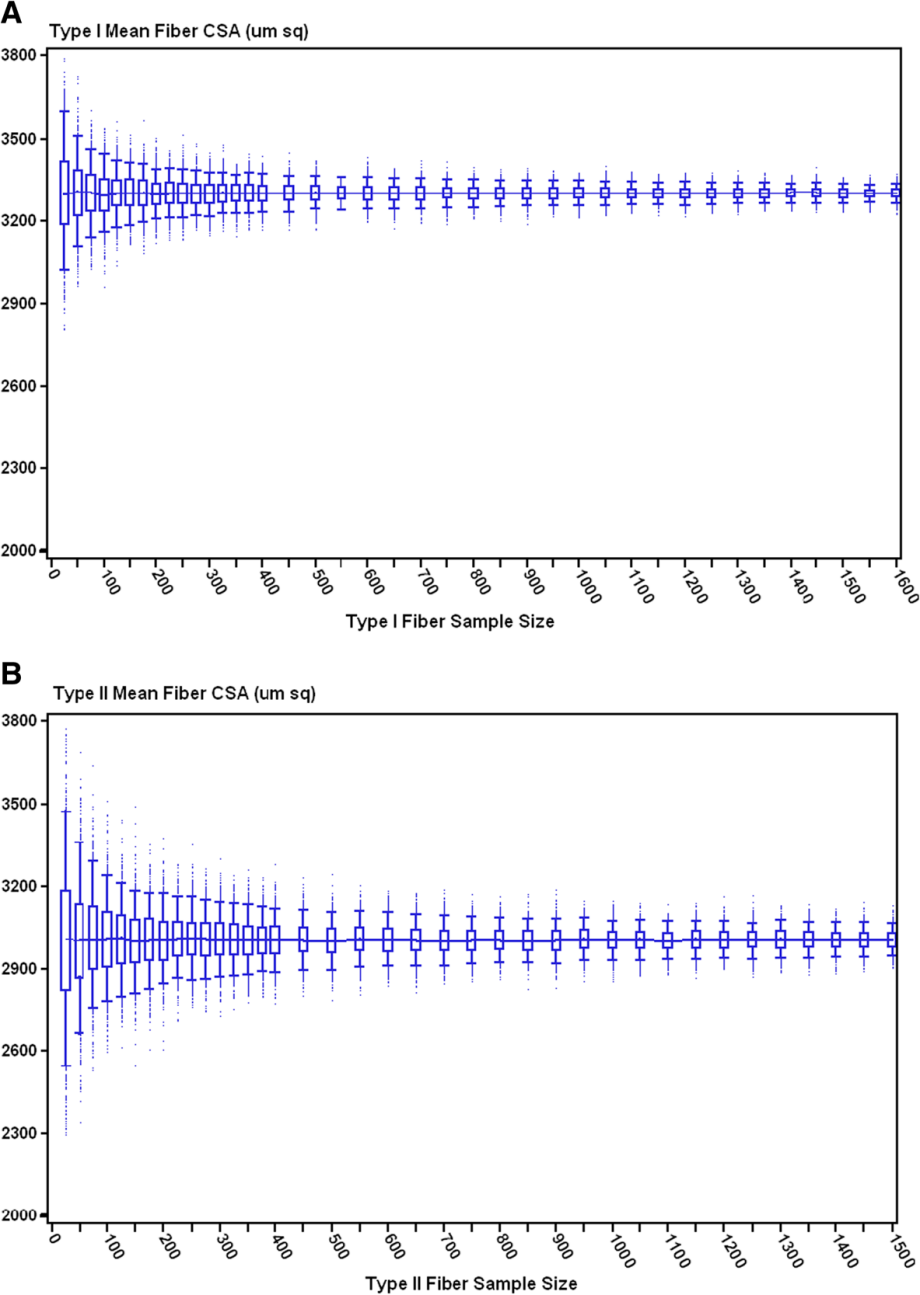
**Box plots of A) bootstrapped mean type I muscle FCSAs by sample size *n *fibers from a single rat soleus muscle; B) bootstrapped mean type II muscle FCSAs by sample size *n *fibers from a single rat EDL muscle.** The line (—) connecting the box plots represents the median; the box represents the 25^th^ and 75^th^ percentiles; the horizontal bars represent the 5^th^ and 95^th^ percentile confidence limits.

**Figure 2:**
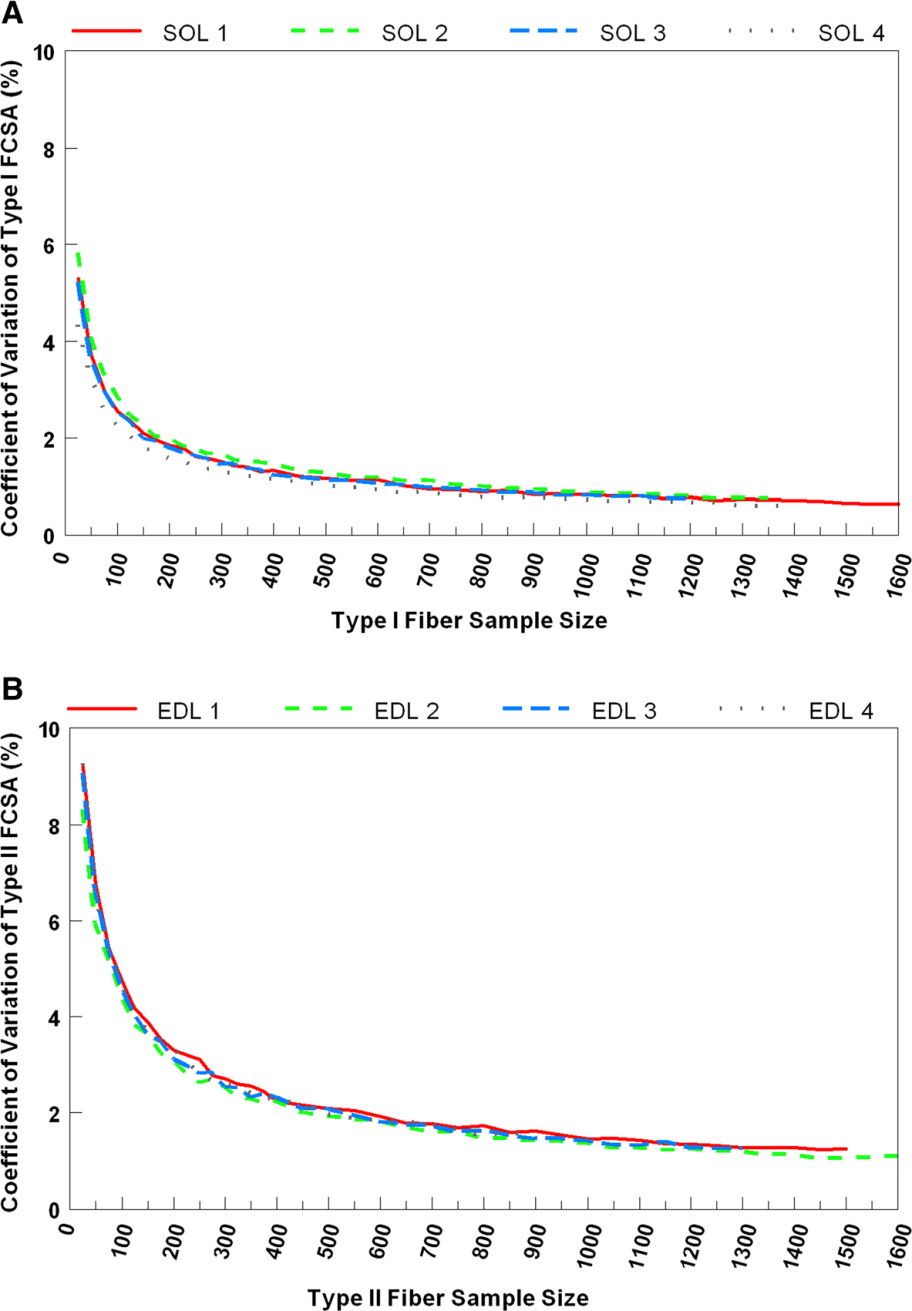
**Line graphs of the coefficient of variation for A) bootstrapped mean type I muscle FCSAs by sample size *n *from the same rat soleus muscle in Figure**1**A; B) bootstrapped mean type II muscle FCSAs by sample size *n *from the same single rat EDL muscle in Figure**1B.

Four soleus and four EDL muscle cross-sections from 4 different rats contained a range of 1000 to 1600 measurable muscle fibers for FCSA. Type I and II FCSAs were obtained from rat soleus and EDL muscles, respectively. Box plots of the bootstrapped mean FCSAs from one rat soleus and EDL are illustrated in Figure 1A and B, respectively. The sampling distribution of bootstrapped replicates of the mean type I and II FCSA reveals that the median of these replicates was similar across the full range of sample sizes starting at 25 fibers measured. Conversely, CVs of the estimates narrowed sharply at sample sizes between 25 and 150 fibers (Figure 2A and B). Beyond sample sizes of 150, the CV continued to decrease at a more gradual rate, plateauing to a steadier level at sample sizes >400. A similar pattern of variability was noted across 4 different samples of each muscle type. In spite of these similarities, the CV values for the bootstrapped mean type I FCSAs were approximately one third lower compared to those for type II FCSAs (Figure 2A and B) regardless of sample size of fibers measured.

### FN

Total FN in 23 muscle cross-sections from 23 rats ranged from 1600 to 2600 fibers. We selected 4 field areas (10x magnification) within the muscle cross-section. FN per field ranged from 100 to 140. We examined the association between FN in 1, 2, 3, and 4 fields and the total FN in 23 rat muscle cross-sections in order to determine whether all 4 fields were needed to better predict total FN in a cross-section. FN from any 3 of the 4 fields was a better predictor of total FN than that from 1 or 2 fields (Table 1), with correlation coefficients ranging from 0.57 to 0.59. FNs from 4 fields did not further improve the correlation coefficient with total FN (r=0.57). Three and 4 of the fields represented approximately 15 and 20% of the total FN, respectively. The regression equation relating the total cross-sectional FN to the 4 field FN was *total FN = 792.25 + 3.08 (four field FN)*.

**Table 1:** The range in correlation of fiber number in 1, 2, 3, and 4 fields with total fiber number in 23 rat muscle cross-sections

**Number of fields**	**Range of correlation coefficients**	**Range of P-values**
1	0.32 to 0.49	0.013 to 0.120
2	0.44 to 0.52	0.009 to 0.035
3	0.57 to 0.59	0.003
4	0.57	0.004

## Discussion

The time and labor involved in estimating muscle FCSA and counting FN in rat skeletal muscle cross-sections can be considerably reduced by evaluating only a subset of the total muscle fibers. Yet, the number of fibers that should be measured to obtain a representative subset has not been well-validated. Our data demonstrate a sharp reduction in the variability of estimates of mean FCSA as the sample size of fibers measured increases, particularly from 25 to approximately 150 fibers. A continued but more gradual improvement in precision of this estimate occurs at sample sizes beyond 150 to approximately 400 fibers. Our study also indicates that the relative standard deviation in mean type I FCSAs at any fiber sample size is about 1/3 lower compared to that in type II, suggesting that fewer type I than type II fibers may need to be measured. Type I muscle fibers from the rat soleus are, thus, more uniform in size than type II muscle fibers from rat EDL. These differences may, in part, be due to the fact that the rat EDL is composed of multiple type II fiber subtypes such as IIa, IIb, and IIx, which differ in size.

Our data also indicate that counting approximately 15% of the muscle fibers in the muscle cross-section provides a reasonable prediction of the total cross-sectional FN in this rat model. We detected similar predictability when the proportion of fibers counted increased to 20% of the total FN.

The strengths of this study include the rigorous statistical procedure used to perform the resampling analysis, the large sample of fibers for each rat with which it was performed, and use of a common laboratory animal model in muscle research. Although our fiber estimates cannot be extrapolated to very aged or diseased rat models with accelerated muscle wasting or, to a lesser extent, middle-aged female rats, our results suggest that the reliability of mean FCSA estimates based on a subset of <150 fibers would be poor due to heterogeneity in fiber size. In addition the methodologies used in this study (bootstrap resampling technique and fiber counting method) to evaluate the reliability of subsets of FCSA and FN could be applied to other animal models to identify more specific threshold levels for FCSA or FN.

## Conclusions

In summary, this report can offer guidance and rationale to future investigators who plan to study muscle fiber size and number in a rat animal model. Our data describe a pattern of improved precision in estimating mean muscle FCSA as the sample size of fibers measured increases, most pronounced from samples of 25 to approximately 150 fibers. We also found that independent of the number of fibers measured for FCSA, estimates of mean type I muscle FCSA from the soleus muscle are generally more precise than type II FCSA from the EDL in this animal model. Regarding FN, FNs from field areas approximating at least 15-20% of the muscle cross-section provide a reasonable prediction of total FN in this rat model.

## Methods

### Experimental animals

F344BN male rats were purchased from the National Institutes of Aging. Male rats were chosen to eliminate the potential confounding effect of hormonal fluctuations on skeletal muscle in this age range. The 23 rats used in these analyses were 23-month-old, were housed individually in plastic cages at 25 degrees C at 12-h-light/12-h-dark cycles, had free access to water, and were part of a metabolic study. Rats were euthanized and hindlimb muscles (soleus and extensor digitorum longus [EDL]) were excised. Soleus and EDL muscles were cut at mid-belly, transversely oriented, and frozen in isopentane liquid nitrogen “slurry” cooled to −158°C for subsequent immunohistochemical analysis. The study was approved by the Institutional Animal Care and Use Committee at Tufts University.

### Immunohistochemistry/histochemistry

**Figure 3:**
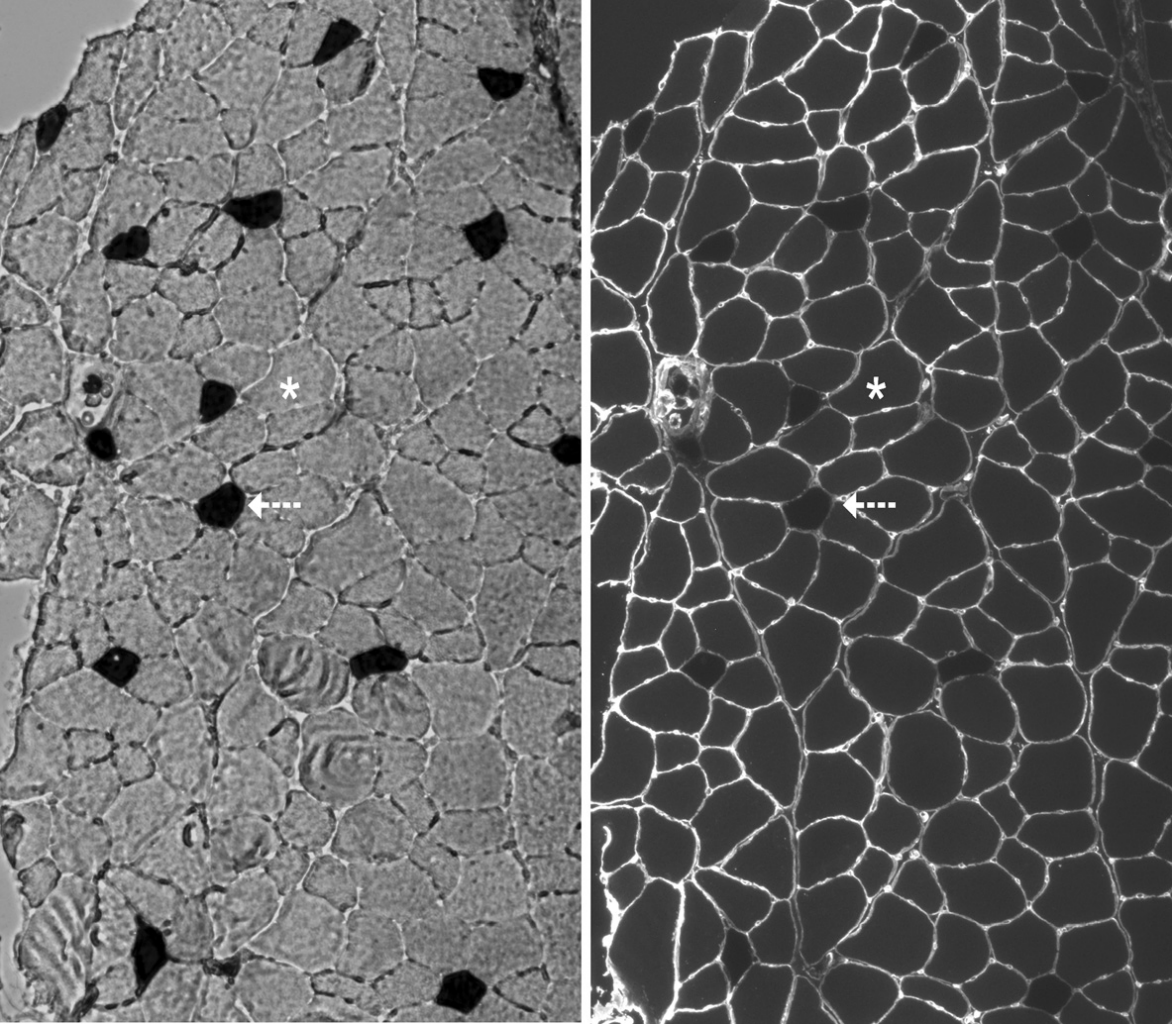
***left panel *Myofibrillar ATPase staining characteristics in an EDL cross-section in 23-month-old F344BN rat [10x magnification].** Dark cells (arrow) indicate positive staining for slow myosin heavy chain (type I) fibers. Light cells (asterisk) indicate fast myosin heavy chain (type II) fibers. *right panel* Immunofluorescent staining for laminin of the same EDL cross-section.

Frozen soleus and EDL muscle samples were cut into 7 μm cross-sections using a cryostat microtome (Leica 1800, Sigma, St. Louis, MI). Cross-sections were immunostained with a rabbit anti-human antibody (IgG; Sigma-Aldrich, Inc.) raised against laminin to facilitate identifying and measuring individual muscle fibers (Figure 3*right panel*). A goat anti-rabbit Alexa Fluor® secondary antibody was used for detection of the laminin primary antibody. Following immunostaining with laminin, the cryosections from frozen soleus and EDL muscle were incubated for myofibrillar ATPase activity after pre-incubation at pH 4.35 to identify type I (heavily stained) and type II (lightly stained) muscle fibers [12]. Soleus muscles were predominantly composed of type I muscle fibers. EDL muscles were predominantly composed of type II muscle fibers (Figure 3*left panel*).

### FCSA measurement

Following the staining protocol, all muscle sections were digitally captured using bright field and fluorescence microscopy with Nikon NIS-AR (3.01). Digital imaging was performed at 10x final magnification. Image processing, including identification of fiber type in all fibers on the muscle cross-section, was performed using Adobe Photoshop® CS3 (Figure 3*left panel*). The circumference of each fiber was outlined using Image J software (v. 1.37) to generate FCSA. Criteria used in the selection of muscle fibers to measure for FCSA included an intact, distinct cell membrane without significant signs of distortion or folding. Elongated fibers indicating an oblique section were excluded. Image analyses were performed by two coauthors (SN and LC). As an assessment of agreement between operators for average FCSA measurement, the mean absolute deviation was less than 0.03.

### FN measurement

Adobe Photoshop® CS3 was employed to manually count all identifiable type I and II muscle fibers within 23 muscle cross-sections from 23 different rats. Criteria to be included in total cross-sectional FN consisted of the ability to distinguish a single fiber and its fiber type (e.g., I or II). Fibers along the periphery that were not complete were also counted, but not measured for FCSA above. We compared total FN to the FN within one to four 10x field areas (1000 x 1500 pixels in size/field) on the cross-section. We limited the number of fields to four, because it was the maximum number of non-overlapping fields that could be fitted on the muscle cross-section at 10x magnification. When possible, the fields were placed in each of the four quadrants of the cross-section that did not have significant tissue artifacts.

### Statistical analysis

A bootstrapping resampling technique was used to determine the variability in sampling distribution of the mean point estimate at varying samples sizes of FCSAs. In the bootstrap procedure, the original dataset of FCSAs of size *N* became a parent population from which samples of size *n* were randomly drawn with replacement with *n*’s ranging from 25 to a maximum of 1600 muscle fibers at increasing increments of 25 (when sample *n*<400) to 50 (when sample *n*≥400). Of note, the upper limit of 1600 fibers in the bootstrap analysis was the maximum number of measurable fibers out of the total number (range 1600 to 2600) in the cross-section that met criteria for FCSA measurement as described above. One thousand bootstrap samples of the mean FCSA were created for each *n* and displayed on a box plot with mean and 5^th^ and 95^th^ percentile confidence limits. The coefficient of variation (CV) of the bootstrapped mean FCSA estimates was also created for each *n* and plotted on a linear graph.

Pearson correlation coefficients and linear regression were used to describe the association between FN based on field area(s) and total FN in the muscle cross-section. Two-sided P values less than 0.05 were considered to indicate statistical significance.

All statistical analyses were conducted with SAS software version 9.2 (Cary, NC).

## Abbreviations

FCSA: Fiber cross-sectional area; FN: Fiber number; F344BN: Fisher 344 x Brown Norway F1 hybrid rat; EDL: Extensor digitorum longus muscle; SOL: Soleus muscle

## Competing interests

No competing interests to disclose.

## Authors’ contributions

LC, BD-H, and RAF designed the study. LC, SN performed the study. LC, BD-H, and RAF contributed study materials and analytic tools. LC and LLP analyzed the data. LC, BD-H, SSH, RAF, and LLP contributed to the writing of the manuscript. All authors approved the manuscript.

## Authors’ information

LC is an Assistant Professor of Medicine at Tufts University and translational investigator in the field of muscle and bone health at Tufts University. SN is an endocrinologist conducting a research postdoctoral fellowship. LLP is a statistician. SSH is a nutritional epidemiologist. RAF is a Professor of Nutrition and Director of the Nutrition, Exercise Physiology and Sarcopenia Laboratory at Tufts University. BD-H is a Professor of Medicine and Director of the Bone Metabolism Laboratory at Tufts University.
